# Healthcare Professionals’ Perspectives on Sepsis Care Pathways—Qualitative Pilot Expert Interviews

**DOI:** 10.3390/jcm14020619

**Published:** 2025-01-18

**Authors:** Lea Draeger, Carolin Fleischmann-Struzek, Jutta Bleidorn, Lena Kannengiesser, Konrad Schmidt, Christian Apfelbacher, Claudia Matthaeus-Kraemer

**Affiliations:** 1 Institute of General Practice and Family Medicine, Jena University Hospital, Friedrich-Schiller-University Jena, 07743 Jena, Germany; 2 Institute of Infectious Diseases and Infection Control, Jena University Hospital, Friedrich-Schiller-University Jena, 07747 Jena, Germany; 3 Center for Sepsis Control and Care, Jena University Hospital, Friedrich-Schiller-University Jena, 07747 Jena, Germany; 4 Institute of Social Medicine and Health Systems Research, Faculty of Medicine, Otto-von-Guericke-University Magdeburg, 39120 Magdeburg, Germany; 5 Institute of General Practice and Family Medicine, Campus Charité Mitte, Charité University Medicine, 10117 Berlin, Germany; 6 Institute of General Practice, Brandenburg Medical School, 14770 Brandenburg, Germany

**Keywords:** sepsis, septic shock, professional care providers, levels of care, expert interviews, qualitative content analysis

## Abstract

**Background/Objectives**: Despite recent decades’ rapid advances in the management of patients with sepsis and septic shock, global sepsis mortality and post-acute sepsis morbidity rates remain high. Our aim was, therefore, to provide a first overview of sepsis care pathways as well as barriers and supportive conditions for optimal pre-clinical, clinical, and post-acute sepsis care in Germany. **Methods**: Between May and September 2023, we conducted semi-structured, video-based, one-to-one pilot expert interviews with healthcare professionals representing pre-hospital, clinical, and post-acute care settings. The interviews were audio-recorded, transcribed verbatim, and analyzed according to the principles of Mayring’s content analysis. **Results**: The eight interviewed professionals identified perceived critical success factors along the entire care pathway with regard to early detection (e.g., disease awareness), early acute treatment (e.g., unknown origin of infection), rehabilitation/aftercare (e.g., availability of primary care actors), and patient transitions within and between sectors (e.g., advance notice of patient arrival). These critical factors comprised: (1) the characteristics of the staff providing care (e.g., available experience), (2) the aids/utilities used (e.g., SOPs), (3) the presentation of the disease (e.g., clear symptoms), (4) the workplace (e.g., high workload), and (5) the cooperation between the staff caring for the patient (e.g., announced and standardized handovers). **Conclusions**: Apart from the specific recommendations that can be derived from the individual factors presented, it can be summarized that all levels of care seem only to be purposeful if providers collaborate and communicate efficiently (i.e., correct triage, multiple-eye principle, transfer management, provision of content-rich medical/discharge letters).

## 1. Introduction

Sepsis is a life-threatening condition characterized by the body’s systemic response to an infection resulting in tissue damage and organ failure. Although the incidence and mortality of sepsis has declined over the last decades, the illness remains a major health burden across the globe with an estimated 50 million patients affected annually [1,2]. Sepsis is a medical emergency, in which the period until recognition and, therefore, the initiation of antimicrobial treatment after symptom onset determines patients’ outcome and prognosis. As lack of or delayed diagnosis impedes or delays the initiation of therapy, screening tools and early warning scores can help to foster a prompt onset of treatment. However, early warning scores can yield a rather low discriminative ability for sepsis, emphasizing the need for more sophisticated instruments [3]. The timeliness of therapy is crucial to the survival of patients as each hour delay increases the likelihood of death [4]. Sepsis is also associated with an increased long-term mortality [5]. Additionally, up to three-quarters of survivors experience at least one newly diagnosed (semi-)permanent physical, psychological, and/or cognitive disability in the first year post-sepsis and newly receive nursing care without prior need [6]. These impairments can persist for years after hospitalization [7], limit the ability to work or return to work [8], and are associated with a substantial decrease in health-related quality of life [5,9]. The multifaceted sets of lasting sequelae require patient-tailored aftercare programs and guidelines to mitigate the patient burden. However, although scientific attention is no longer focused solely on the acute event but instead has gradually expanded its scope to measures of rehabilitation [10,11], widely available follow-up programs and facilities to ease survivors’ impairments are currently scarce [12].

Due to the condition’s complexity, septic patients usually pass through several medical sectors and disciplines (i.e., emergency medicine, intensive care medicine, surgical specialties, internal medicine, neurology, rehabilitative medicine, and family medicine) creating several odds for interface and handover situations. A clinical handover can be defined as the exchange of information about a joint patient between health professionals that is attended by a shift in control over and/or responsibility for the patient from one (group of) health professional(s) to another (group) occurring either temporarily or permanently [13,14]. Along these handover passages, vital information for optimal patient management can be lost, which renders holistic management tailored to the individual patient needs more difficult [15,16].

Although several international studies have shed light on the barriers and facilitators to timely and adequate sepsis care, German data on that topic are lacking. In general, there are little qualitative data on the factors influencing timely diagnosis and treatment as well as the successful handover processes of septic patients in the German healthcare system, especially before and after hospitalization. In particular, the factors contributing to successful rehabilitation and follow-up of sepsis survivors have not yet been conclusively elucidated. To address these gaps, our research team conducted two focus group studies to gain a holistic view on barriers and supportive conditions in pre-hospital, clinical, and rehabilitative settings as well as their interfaces. To prepare for the focus groups content-wise, preceding expert interviews were carried out. The expert interviews as well as the insights gained from them are outlined in this article.

## 2. Materials and Methods

### 2.1. Design

We conducted qualitative semi-structured interviews with German experts in sepsis care. The interviews were conducted as upstream interviews within the framework of the project “AVENIR—Improving the care of sepsis patients: Analysis of care pathways, experiences, and needs of patients with and after sepsis” [17]. Their purpose was to inform the interview guidelines for two consecutive series of focus groups. Specifically, the interviews were conducted prior to an in-depth focus group study to enhance familiarity with the subject matter beyond a mere literature review and to develop a topic guide for the subsequent focus groups.

In qualitative research, expert interviews serve as a method to gather in-depth, specific insights into a narrowly defined area of interest. The focus lies on the specialized knowledge of the experts. While the definition of “expert” can vary, experts are typically identified by their knowledge of the predefined field of interest, their unique position in society, or their status [18]. In this case, the interviews aimed to capture the perspectives of professional healthcare stakeholders on sepsis care in Germany, with a particular focus on identifying (a) factors that contribute to optimal acute and long-term care and (b) factors that hinder it. Of interest were (1) early detection, (2) acute treatment, (3) rehabilitation and aftercare, and finally, (4) transitions in care. Using this method, we were able to obtain expert input from providers working directly at the patient’s bedside. As the expert interviews follow an observational approach, we cannot make any statements about the actual impact of the factors on patient outcomes. Lastly, the reporting standard SRQR [19] was used to provide transparency into the research process (see Supplementary SA).

### 2.2. Sample

For the present interviews, a multi-professional and multi-disciplinary sample of professional sepsis care actors was selected aiming for maximum variation. Inclusion criteria were current medical occupation (such as physician or emergency paramedic) within the German healthcare system as well as active management of septic patients or sepsis survivors regardless of participants’ medical faculty. We recruited participants from the pre-clinical (emergency medicine), clinical (intensive care) as well as post-clinical (rehabilitation medicine and general medicine) sectors. The exclusion criteria were retirement and no role in sepsis management in the current medical activities. Recruitment was informed by in-house online databases of Thuringian family physicians with willingness to participate in scientific studies, website information of relevant hospitals as well as personal contacts. Interview participants were contacted directly by phone or email and were provided with written detailed information about the purpose of the study. Reasons for non-participation were lack of time or perceived lack of sepsis focus in one’s occupation. Written informed consent was obtained from all interview partners. The protocol and the consent form were fully approved (2023-2973-Bef) by the Friedrich-Schiller-University Ethics Committee, Jena, Germany.

### 2.3. Data Collection and Analysis

Semi-structured, video-supported, one-to-one interviews took place between May 2023 and September 2023 and were led by one interviewer trained in qualitative research methods (LD). Interviewees participated from their workplace or private environment. Interviews were guided by a thematic topic guide with open-ended questions, developed by means of a literature review (review currently under consideration) to explore key topics related to every phase of sepsis care: recognition, treatment, rehabilitation/aftercare, and transitions of care; see Supplementary SB. The selection of themes was tailored to the participant’s scope of therapy (e.g., family physicians were not asked about their perspective on intensive care treatment). To ensure comprehensibility, the topic guide was critically discussed within two research groups consisting of scientific staff of the Friedrich-Schiller-University in Jena, the Otto-von-Guericke University in Magdeburg, and the Global Sepsis Alliance in Berlin, Germany, and pilot-tested once with the first interview participant. The pilot interview was included in the analysis, as no changes were made to the guideline afterward. Interviews were recorded using digital audio recorders, transcribed verbatim using the software f4transcripts (version 3) and MAXQDA (version 2022), anonymized, and analyzed with MAXQDA. To parse the transcripts and to identify grouped qualitative codes, the Qualitative Content Analysis according to Mayring’s procedure of content structuring [20] was used. Mayring describes three distinct analytical procedures, which we used in combination: (1) summary, (2) explication, and, as already mentioned, (3) structuring. Within the first procedure, we reduced the material while retaining the essential content by broad paraphrasing and condensing. Within the second procedure, the material was annotated by developing explicatory paraphrases in consideration of the relevant context. Lastly, definitions of codes were developed and agreed upon, prime examples were extracted, and definition of coding rules were specified. Main codes were built deductively and subcodes were built inductively. To ensure rigor of content analysis and intersubjectivity, two independent coders (LD and CMK) coded all transcripts and reviewed all codes. We concentrated on codes mentioned by ≥2 participants (at least 25% of participants) for the analysis. Within the expert interviews, participants framed a factor that affects the quality of sepsis care either negatively as a barrier or positively as a facilitator at times, leading to barriers and facilitators with matching content. Factors that were described as facilitators in the majority of cases are also listed as facilitators in the following. The same applies to barriers. Yet, barriers can be phrased as facilitators when inverted and vice versa. As the interviews were conducted in German, the interview guide, the codes as well as the quotes were translated from German to English for this manuscript.

## 3. Results

The mean duration of the eight interviews conducted was 35 min (range 21 to 60 min). Thus, 280 min (4.7 h) of interview material could be collected in total. Interviewees were located in five federal states of Germany (Bavaria, Berlin, Mecklenburg–West Pomerania, Saxony, and Thuringia). Participants’ mean age was 47 years (range 26 to 59 years). Interviewees’ mean work experience in their field was 22 years (range 3 to 34 years). Three participants worked in the pre-clinical (emergency medical service and emergency department), two in the clinical (intensive care unit), and three in the post-acute setting (rehabilitation clinic and primary care). Participants’ professions and further characteristics are listed in Table 1.

All participants specified that sepsis care plays a key role in their everyday practice, ascribing sepsis care a very high relevance in their professional work.

*“The treatment of sepsis is really part of my daily routine—or of severe infections with septic courses. I’m confronted with it every day, so to speak […]. So the topic of infections and septic courses is what we deal with the most, so to speak”*.

(participant No. 5)

Furthermore, some participants placed the critical illness of sepsis in direct comparison with other critical illnesses and highlighted differences in terms of the clinical picture and management.

*“In the case of an ST-segment elevation myocardial infarction, you write an ECG, it’s low-threshold, it can be done quickly, any nurse, any doctor can do it […]. The diagnosis is clear, he [the patient] goes into the cardiac catheter, receives his initial therapy as medication, and then it’s a done deal, I say. But with sepsis, it’s so difficult […]”.*

(participant No. 2)

The performance of management was described as being generally too slow.

*“In the case of sepsis, which is in no way inferior [to a stroke] in terms of mortality, it is totally interesting that perhaps/perhaps, this is a guessing game, but because the patients may not appear so acutely ill, an incredible amount of time is actually wasted. So I often notice that when you say the keyword “sepsis” or report a patient with sepsis in the transferring hospital, then […] there’s no difference in the pace of action or in the/yes, the pace of work from sepsis to renal colic or to a fracture of the lower leg”.*

(participant No. 1)

Moreover, the family practitioners described the joy felt at their patient’s survival following sepsis.

*“I’m always happy when such seriously ill patients survive and when they leave the hospital alive at all […]. And then I’m happy when I can continue to care for them and when they gradually get better again. And that’s what we do our work for. I’m always happy when I’ve made a good assessment, when I’ve admitted the patient and I’ve said: “Man, you’re seriously ill, you need to go to the hospital immediately“ […]. That [survival] doesn’t happen very often”.*

(participant No. 7)

Additionally, some participants depicted the psychological aftereffects that patients frequently suffer from after the acute illness.

*“Many are in need of help and even if the patients are still physically able to live independently and alone at home again, many suffer from secondary illnesses such as reduced resilience, traumatic disorders, anxiety disorders, so that […] there is also a great need for action here”.*

(participant No. 6)

For better readability, the quotes best representing our results below are listed in Supplementary SC.

### 3.1. Facilitators to the Early Recognition of Sepsis and Septic Shock

**Figure 1:**
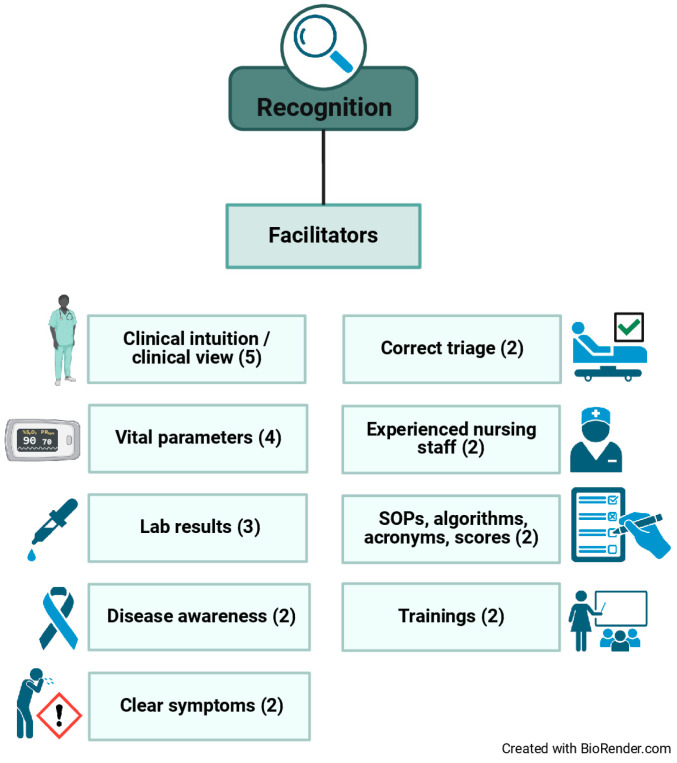
Facilitators to the early recognition of sepsis and septic shock.

The most frequently mentioned facilitator was the professional’s clinical intuition/view to detect sepsis. Further factors instrumental in identifying sepsis were the usage of vital parameters and the enlistment of lab results. Providers’ awareness of the critical illness sepsis as such, a patient showing clear symptoms, correctly triaged patients, experienced nursing staff, the application of Standard Operating Procedures (SOPs), sepsis algorithms, acronyms, and scores as well as trainings in detecting sepsis were also discussed as helpful to the early identification of sepsis and septic shock. The conditions that were perceived to enhance the process of diagnosis are depicted in Figure 1. The numbers in parentheses indicate the number of interviewees who invoked the respective facilitator during the interview.

### 3.2. Barriers and Facilitators to the Timely Treatment of Sepsis and Septic Shock

**Figure 2:**
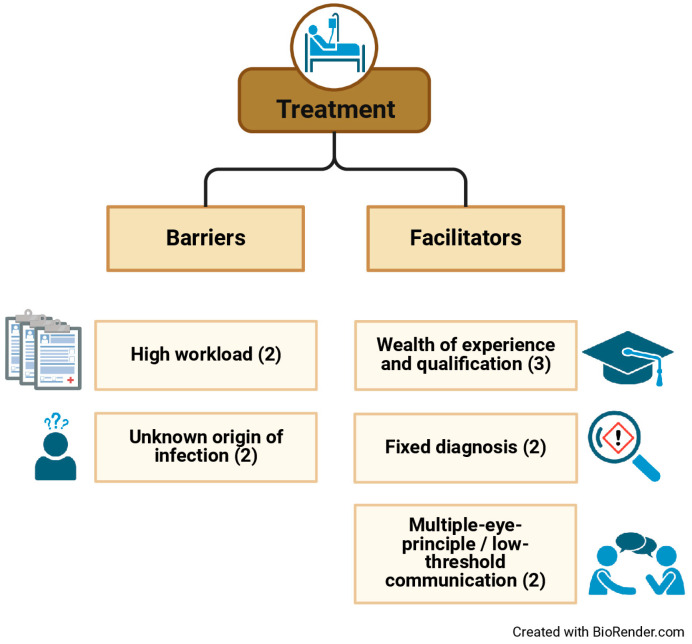
Barriers and facilitators to the timely treatment of sepsis and septic shock.

The most commonly cited barriers to promptly initiated treatment were a high workload and an unknown origin of infection. The main contributing factor was the wealth of experience and qualification of providers. Further cited conditions making timely and appropriate treatment more likely were a fixed diagnosis of sepsis and, at last, patient surveillance by multiple providers who communicate in a low-threshold manner. Figure 2 illustrates the barriers that were perceived to hinder and the facilitators that were perceived to foster prompt treatment. The numbers in parentheses indicate the number of interviewees who mentioned the respective factor during the interview.

### 3.3. Factors That Influence the Adequate Rehabilitation/Aftercare of Sepsis Survivors

**Figure 3:**
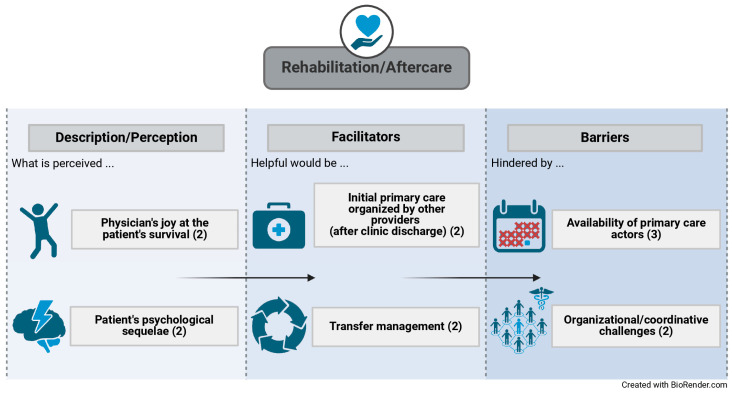
Factors that influence the adequate rehabilitation/aftercare of sepsis survivors.

As already mentioned, the providers interviewed described the joy felt at their patient’s survival following sepsis but also the psychological aftereffects that patients frequently suffer from after the acute illness. Providers rate the prompt organization of initial primary care (e.g., medical aids and nursing service) for the time right after hospital discharge as beneficial to successful rehabilitation and aftercare. Moreover, to ensure adequate rehabilitation and aftercare, respondents considered the implementation of transfer management from the inpatient to the post-acute or outpatient sector as helpful. However, the lack of availability and accessibility of primary care actors (e.g., psychologists and physiologists) and the organizational and coordinative challenges of, for instance, scheduling appointments of different care actors are perceived to hinder the process of felicitous rehabilitation and aftercare. The factors that were perceived as conducive as well as deleterious to a successful rehabilitation or follow-up process, respectively, are portrayed in Figure 3. The numbers in parentheses display the number of interviewees who pointed out the respective factor during the interview.

### 3.4. Factors That Influence the Successful Handovers of Septic Patients and Sepsis Survivors

**Figure 4:**
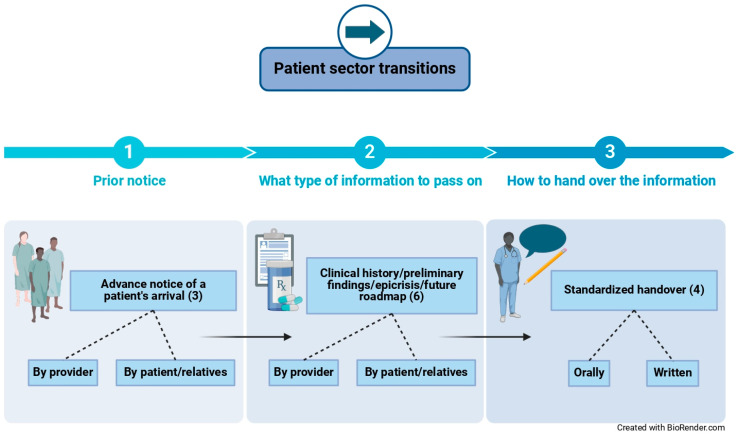
Factors that influence the successful handovers of septic patients and sepsis survivors.

When asked about supportive conditions during patient handovers, providers explained to value the prior notification of the patient’s imminent arrival (either by the upstream providers or the patients and relatives themselves) in order to be able to prepare for the patient’s admission or visit accordingly. For the handover process itself, providers stated to appreciate the disposal of patient information on, for example, anamnesis (e.g., current medication plan, allergies), preliminary findings, epicrisis, and recommended actions for subsequent management (either by the upstream providers or the patients and relatives themselves if possible). Finally, participants declared to prefer handovers in a standardized form that includes the oral and written dissemination of relevant patient information. Figure 4 visualizes the factors that providers perceived as contributing to successful patient handovers throughout the interfaces of units and sectors that patients pass through. The numbers in parentheses report the number of interviewees who cited the respective factor during the interview.

## 4. Discussion

The interviews conducted identified a variety of factors described to affect the quality of sepsis care by experts involved in sepsis care within the four closely intertwined care segments recognition, acute treatment, rehabilitation, and aftercare as well as patient transitions. The most prominent factors discussed by our interviewees operating in the pre-clinical, clinical as well as post-acute sectors can be broadly subsumed under five domains: the individual provider (clinical intuition/view, sepsis awareness, experience and qualification), the aids/utilities used (vital parameters, lab results, SOPs/algorithms/acronyms/scores, trainings), the disease presentation (clear symptoms, unclear origin of infection), the workplace (workload), and finally, the level of collaboration and communication between providers (correct triage, fixed diagnosis, multiple-eye principle/low-threshold communication, organization of initial primary care, transfer management, availability of primary care actors, organizational/coordinative challenges, prior notice of patient arrival, anamnesis/preliminary findings/epicrisis/future roadmap, standardized handovers).

International literature that covers influencing factors of processes of sepsis recognition and treatment describes parameters similar to those depicted by our German interviewees (review currently under consideration). As already mentioned, early recognition and treatment of septic patients are of utmost importance as delayed medical measures are associated with adverse health outcomes such as death and higher morbidity [21,22]. However, due to high patient volumes, necessary divided attention of medical staff, and other contextual factors, the effective management of septic patients can be demanding [23]. For that reason, the Surviving Sepsis Campaign [4] pointed out sepsis alert systems as potential instruments to counteract this challenge. A systematic review and meta-analysis from 2024 with 22 included studies investigated the link between sepsis alert systems and the risk of mortality as well as the adherence to the sepsis bundle with septic patients treated in the emergency department. The authors found that sepsis alert systems are, on the one hand, associated with a reduced risk of mortality and, on the other hand, with a better adherence to the sepsis bundle—not only in terms of comprehensiveness but also speed. This association was stronger in electronic than in non-electronic alert systems. These results underline the potential value of the introduction and integration of electronic sepsis alert systems into clinical practice in the emergency department and potentially beyond. However, the sensitivity for detecting sepsis varied highly between studies [24].

Especially in Germany, there is still little knowledge about professionals’ requirements to ensure smooth patient transitions and to provide structured sepsis rehabilitation and aftercare. To our knowledge, the first and only German multi-professional qualitative study exploring conducive and adverse factors on optimal sepsis care among and between several interfaces of care was undertaken by Matthaeus-Kraemer and colleagues [25]. However, the results of Matthaeus-Kraemer et al. [25] are limited by the fact that they only included hospital staff.

Regarding patient transitions, the notification of a patient’s arrival and a standardized handover (oral and written) including all the relevant medical information about the prior and ideal prospective course of treatment were recommended to reduce the risks inherent to handovers by our interviewees. When caring for critically ill septic patients, the hand-off of patients can take place at a multitude of times within a course of treatment. Not only larger-scale responsibility transfers that happen when a patient is transferred between healthcare sectors, hospitals, or wards are classified as handovers. A transfer of responsibility can also occur for a short period, for instance, when a shift ends and when providers are appointed elsewhere or pause work to, e.g., have lunch [26]. If delivered well, clinical handovers can foster continuous care, the accumulation of crucial knowledge about the patient and, therefore, patient-tailored treatment decisions, conversely mitigating treatment errors. However, if not delivered well, high standards of care cannot be maintained. More specifically, a clinical handover can be considered “a high risk scenario for patient safety with dangers of discontinuity of care, adverse events and legal claims of malpractice” [27]. A study by Leonard and colleagues [28] suggests that up to 70 percent of adverse medical events in hospitals can be traced back to breakdowns in communication, making it a core problem of healthcare services. The growing awareness of handover pertinence has directed organizational attention toward the prioritization and optimization of handover processes, such as by the British Medical Association, the Australian Medical Association, and the WHO. To summarize, the Australian Medical Association [13] denotes that handovers rely on both systemic and individual attention and approves that the continuity of relevant patient information determines patient safety. Problematically, handovers can be highly variable, are often performed informally, and are prone to errors [29]. Beyond, in the year of 2009, Cohen and Hilligoss [14] criticized that English literature on hospital handovers inconclusively defined the meaning of standardization. In response to gaps like these, structured and standardized handover tools such as the now widely used and well-studied acronym ISBAR were developed. The acronym stands for introduction, situation, background, assessment, and recommendation [30]. The framework can be used for a variety of clinical contexts and interactions, for short and long-term as well as intra- and interprofessional transfers. The ISBAR template is targeted on the collection of information needed to obtain a patient’s clear clinical picture and on the documentation of pending tasks [30]. Nevertheless, research from Australia denotes that too much duplication and/or irrelevant information and the omission of critical detail are persistent risks to handover quality [31,32]. Additionally, as technological and medical innovations within increasingly complex healthcare systems progressively pass, continued efforts are needed to make the septic patient journey safer.

Another important finding that evolved in our upstream interviews is that all factors depicted regarding the process of rehabilitation and aftercare, relate to the multi-professional approach and its provider coordination needed to set up sepsis follow-up appropriately. Providers interviewed alluded to the organization of initial primary care for the time right after hospital discharge and the implementation of transfer management from the inpatient to the post-acute or outpatient sector as advantageous. According to our participants interviewed, the low availability and accessibility of primary care actors as well as the organizational and coordinative challenges are barriers to successful rehabilitation and aftercare. Current evidence shows that sepsis survivors profit from the following elements of aftercare: screening for new physical, cognitive, and psychological ailments with the referral to a specialist, screening and altering medication plans if needed, and lastly, remaining vigilant toward treatable conditions that often result in re-hospitalization, such as infection [9]. Additionally, the provision of palliative care might be appropriate for survivors who face increasing deterioration [9]. Although sepsis sequelae comprise a variety of physical, cognitive, and psychological symptoms, German rehabilitation therapies typically focus on physical impairments (physical therapy, occupational therapy, and speech and language therapy) rather than on cognitive or psychological impairments. Therapies geared to issues of fatigue, pain, weaning of ventilation, and mental health are way less common [33]. Furthermore, survivor satisfaction with hospital services that should prepare patients for the time after discharge is mixed. Patients reported dissatisfaction with the availability of psychological counseling after discharge, post-sepsis education, and social service support [34]. Beyond, ICU survivors tend to increasingly consult their family practitioner in the year after the critical illness and hospital discharge [35]. Therefore, despite the multi-professional approach needed to meet the needs of survivors, the family practitioner will most likely be the main contact for outpatient care coordination. However, difficulties in information sharing between family practitioners and ICU staff during and after a patient’s incident of critical illness and, therefore, in transfer management have been reported [36,37]. The absence of transfer management limits primary care providers’ informedness about a patient’s critical illness and its context, which complicates the already complex provision of follow-up [37]. To support family practitioners in the endeavor to identify and meet the needs of survivorship, e.g., primary care management interventions might be promising to reduce survivors’ late-onset mental health issues [38]. However, such outpatient interventions are scarce, and little evidence exists for their effectiveness in improving different dimensions of frailty. Also, limited clinical trial evidence for the positive effects of interventions applying case management for critically ill patients exists [39,40]. To conclude, as only half of the sepsis survivors recover completely within two years after the acute episode [9], more research is needed on how to conceptualize structured aftercare interventions to master the immense amount of survivors who will seek medical help—presumably in primary care.

### Strengths and Limitations

One notable strength of our study is the multi-professional and multi-disciplinary nature of the sample, which allowed us to incorporate a variety of perspectives from different stakeholders in sepsis care. This diversity enhances the comprehensiveness and applicability of the findings. Methodological rigor was ensured by involving two independent researchers in coding the data and systematically reviewing the coding, thereby increasing the reliability and validity of the analysis. Additionally, the use of digital interviews, conducted via a conference system software, provided logistical advantages. This approach enabled us to recruit participants from across the entire country, thereby ensuring geographical variety and representation in the sample. Digital interviews also facilitated scheduling flexibility, which likely contributed to participant accessibility and engagement.

Despite these strengths, the study has certain limitations. One of these is the relatively small sample size. The small number of cases might be due, among other things, to the high workload in medical care. However, given that the primary purpose of the interviews was to prepare the content for the subsequent focus group discussions, the sample size was deemed appropriate for this exploratory pilot phase. The aim was not to achieve data saturation but rather to generate preliminary insights and to inform the development of the focus group topic guide.

Another limitation arises from the digital nature of the interviews. While this approach expanded the geographical reach of the study, it may have constrained the depth of information shared by participants. In-person interviews might have fostered a more personal rapport, potentially encouraging more detailed or candid responses. However, it is also conceivable that some people speak more freely in the digital space precisely because they do not feel intimidated by a direct one-on-one-interview.

Moreover, the lack of anonymity during the interviews could have introduced social desirability bias, as participants were directly reporting to the interviewer. This may have influenced their responses, leading them to present views they perceived as more favorable.

Finally, the possibility of volunteer bias should be acknowledged. The study relied on participants who actively agreed to take part, which means the sample may not fully represent the perspectives of those who were unwilling or unable to participate. As a result, the findings may disproportionately reflect the views of individuals who were more motivated or interested in the topic.

In conclusion, while the methodological strengths of the study provide robust and valuable insights into sepsis care, the noted limitations highlight the need for cautious interpretation of the findings. These insights primarily serve to inform and refine subsequent phases of the research.

## 5. Conclusions

Apart from the specific recommendations that can be derived from the individual factors presented, it can be summarized that all levels of care seem only to be purposeful if providers collaborate and communicate efficiently (i.e., correct triage, multiple-eye principle, transfer management, provision of content-rich medical/discharge letters). As directly targeting critical success factors of optimal care may maximize treatment success, future work should advance change strategies to current practice. Also, further research should focus on the proposition and assessment of solutions for the challenges mentioned. Downstream focus groups conducted by the research team of AVENIR intend to bring this proposition to the fore.

## Figures and Tables

**Table 1:** Sample characteristics.

No.	Gender	Profession	Specialty	Years of Practice	Field; Hospital Size; Care Level	Location
1	male	emergency paramedic	-	3 years	emergency medical services	medium-sized urban area ^3^
2	female	physician	internal medicine; clinical acute and emergency medicine; emergency medicine	15 years	emergency department; 600 to 700 beds; care level 3: specialized/ centralized care	small-sized urban area ^2^
3	male	physician	general medicine; anesthesiology; clinical acute and emergency medicine; emergency medicine	25 years	emergency department; 600 to 700 beds; care level 3: specialized/ centralized care	small-sized urban area ^2^
4	male	intensive care nurse	-	10 years	intensive care unit; 900 to 1000 beds (university hospital); care level 4: maximum care	medium-sized urban area ^3^
5	male	physician	internal medicine; intensive care medicine	34 years	intensive care unit; 100 to 200 beds; care level: not classifiable	megacity ^4^
6	male	physician	neurology; intensive care medicine	23 years	intensive care unit; over 1000 beds; care level 3: specialized/ centralized care	rural area ^1^
7	female	physician	internal medicine	29 years	private practice	rural area ^1^
8	female	physician	general medicine; palliative medicine	34 years	private practice	rural area ^1^

^1^ up to 4.999 inhabitants. ^2^ between 5.000 and 19.999 inhabitants. ^3^ between 20.000 and 99.999 inhabitants. ^4^ 1,000,000 inhabitants or more.

## Data Availability

The participants of this study did not give written consent for their audio data to be shared publicly, so supporting data are not available. The anonymized audio transcripts are available from the corresponding author upon reasonable request.

## Supplementary Materials

The following supporting information can be downloaded at: https://www.mdpi.com/article/10.3390/jcm14020619/s1, Supplementary SA: Standards for reporting qualitative research; Supplementary SB: Interview guide for expert interviews, Supplementary SC: Example quotations.
