# Supplementary material for: Healthcare Professionals’ Perspectives on Sepsis Care Pathways—Qualitative Pilot Expert Interviews
Source: J Clin Med. 2025 Jan 18;14(2):619. doi: 10.3390/jcm14020619 (PMC11766067; doi:10.3390/jcm14020619)
Supplement: Supplementary file 1 [file jcm-14-00619-s001.zip › Supplementary SA_SRQR.pdf]

Supplementary SA: Standards for reporting qualitative research

Based on the SRQR guidelines (O'Brien BC, Harris IB, Beckman TJ, Reed DA, Cook DA. Standards for reporting qualitative research: a synthesis of recommendations. Acad Med. 2014;89(9):1245-1251) for the article:

Healthcare professionals' perspectives on sepsis care pathways – qualitative pilot expert interviews

***Supplementary Table S1.* Standards for reporting qualitative research (SRQR)**

Chapter	Reporting item	Location in text
**Title**	**#1:** Concise description of the nature and topic of the study identifying the study as qualitative or indicating the approach (e.g., ethnography, grounded theory) or data collection methods (e.g., interview, focus group) is recommended	**#1** Title
**Abstract**	**#2:** Summary of the key elements of the study using the abstract format of the intended publication; typically includes background, purpose, methods, results, and conclusions	**#2** Abstract
**Introduction**	**#3 Problem formulation:** Description and significance of the problem / phenomenon studied: review of relevant theory and empirical work; problem statement<br><br>**#4 Purpose or research question:** Purpose of the study and specific objectives or questions	**#3** Introduction Paragraph 1-3<br><br>**#4** Introduction Paragraph 3 & Design
**Methods**	**#5 Qualitative approach and research paradigm:** Qualitative approach (e.g., ethnography, grounded theory, case study, phenomenolgy, narrative research) and guiding theory if appropriate; identifying the research paradigm (e.g., postpositivist, constructivist / interpretivist) is also recommended; rationale. The rationale should briefly discuss the justification for choosing that theory, approach, method or technique rather than other options available; the assumptions and limitations implicit in those choices and how those choices influence study conclusions and transferability. As appropriate the rationale for several items might be discussed together.<br><br>**#6 Researcher characteristics and reflexivity:** Researchers' characteristics that may influence the research, including personal attributes, qualifications / experience, relationship with participants, assumptions and / or presuppositions; potential or actual interaction between researchers' characteristics and the research questions, approach, methods, results and / or transferability<br><br>**#7 Context:** Setting / site and salient contextual factors; rationale	**#5** Design<br><br>**#6** Researcher characteristics and reflexivity in Appendix C<br><br>**#7** Data collection and analysis<br><br>**#8** Design & Sample

	**#8 Sampling strategy:** How and why research participants, documents, or events were selected; criteria for deciding when no further sampling was necessary (e.g., sampling saturation); rationale<br><br>**#9 Ethical issues pertaining to human subjects:** Documentation of approval by an appropriate ethics review board and participant consent, or explanation for lack thereof; other confidentiality and data security issues<br><br>**#10 Data collection methods:** Types of data collected; details of data collection procedures including (as appropriate) start and stop dates of data collection and analysis, iterative process, triangulation of sources / methods, and modification of procedures in response to evolving study findings; rationale<br><br>**#11 Data collection instruments and technologies:** Description of instruments (e.g., interview guides, questionnaires) and devices (e.g., audio recorders) used for data collection; if / how the instruments(s) changed over the course of the study)<br><br>**#12 Units of study:** Number and relevant characteristics of participants, documents, or events included in the study; level of participation (could be reported in results)<br><br>**#13 Data processing:** Methods for processing data prior to and during analysis, including transcription, data entry, data management and security, verification of data integrity, data coding, and anonymisation / deidentification of excerpts<br><br>**#14 Data analysis:** Process by which inferences, themes, etc. were identified and developed, including the researchers involved in data analysis; usually references a specific paradigm or approach; rationale<br><br>**#15 Techniques to enhance trustworthiness:** Techniques to enhance trustworthiness and credibility of data analysis (e.g., member checking, audit trail, triangulation); rationale	**#9** Sample & Ethics (back material)<br><br>**#10** Data collection and analysis<br><br>**#11** Data collection and analysis & Appendix A<br><br>**#12** Sample, Results Paragraph 1 & Table 1<br><br>**#13** Data processing in Appendix C<br><br>**#14** Data collection and analysis<br><br>**#15** Data collection and analysis
**Results/findings**	**#16 Syntheses and interpretation:** Main findings (e.g., interpretations, inferences, and themes); might include development of a theory or model, or integration with prior research or theory<br><br>**#17 Links to empirical data:** Evidence (e.g., quotes, field notes, text excerpts, photographs) to substantiate analytic findings	**#16** Results & Syntheses and interpretation in Appendix C<br><br>**#17** Data availability statement (back material) & Appendix B
**Discussion**	**#18 Intergration with prior work, implications, transferability and contribution(s) to the field:** Short summary of main findings; explanation of how findings and conclusions connect to, support, elaborate on, or challenge conclusions of earlier scholarship; discussion of scope of	**#18** Discussion<br><br>**#19** Strengths and limitations

	application / generalizability; identification of unique contributions(s) to scholarship in a discipline or field<br><br>**#19 Limitations:** Trustworthiness and limitations of findings	
**Other**	**#20 Conflicts of interest:** Potential sources of influence of perceived influence on study conduct and conclusions; how these were managed	**#20** Conflict of interest (back material)
	**#21 Funding:** Sources of funding and other support; role of funders in data collection, interpretation and reporting	**#21** Acknowledgements (back material)

#6 Researcher characteristics and reflexivity

The interviewer (LD) conducted the interviews as part of her PhD program in health services research after her master's degree in psychology. She had not met any of the participants prior to the interviews. Prior contact was undertaken exclusively via phone call and/or e-mail. The interviewer already conducted qualitative interviews in the past and underwent training in how to behave as an interviewer. When the interview phase started and ended, she had worked in the field of health services research for less than three years and therefore qualifies as an early career researcher. Due to the information imbalance between the experts and the psychologist interviewer, content-related questions on the part of the interviewer arose during the interview. When terms or concepts were not familiar to the interviewer, explanations were requested. Beyond, the interviewer considered how existing interprofessional dynamics (medical provider vs. psychologist/researcher) were shaping the interactions, e.g., whether the interviewees would have disclosed different information with an interviewer with medical experiences in patient care. Regarding the analysis, the deductive coding was influenced by an upstream literature review and the structure of the topic guide. The transcripts were additionally analyzed by a second researcher (CMK) with a sociological as well as medical background and extensive qualitative research experience.

#13 Data processing

All recordings were irrevocably deleted from the recording devices as well as from the interviewer's access restricted internal institute drive after transcription. The anonymized transcripts (with removal of all personal references) are stored in the access-restricted internal institute drive for 10 years after project completion in accordance with the guidelines of good scientific practice.

#16 Syntheses and interpretation

Due to the abundance of codes that emerged during the interviews, the final presentation of results focused on the barriers/success factors mentioned at least twice in order to condense the data. In the medical context, it is customary to reduce the data and quantify it to a certain extent in order to provide readers with an overview of the analysis results in an article with a limited word count.
