# Supplementary material for: Healthcare Professionals’ Perspectives on Sepsis Care Pathways—Qualitative Pilot Expert Interviews
Source: J Clin Med. 2025 Jan 18;14(2):619. doi: 10.3390/jcm14020619 (PMC11766067; doi:10.3390/jcm14020619)
Supplement: Supplementary file 1 [file jcm-14-00619-s001.zip › Supplementary SB_Interview_guide_for_expert_interviews.pdf]

Supplementary SB: Interview guide for expert interviews

Getting started

1. What significance/relevance does sepsis care have in your everyday work?

2. When you think of the care of septic patients in your everyday work, what is the first thing that comes to mind?

Recognition/diagnosis

We are interested in what experience you have had with the recognition or diagnosis of sepsis/septic shock in your everyday professional life. Note: The treatment of sepsis will be discussed separately. For this moment, please focus exclusively on recognition.

3. How do you perceive and how would you describe the process of recognizing and diagnosing sepsis?

4. What makes it easier for you personally in your everyday work to recognize septic patients as septic at an early stage? Please explain.

5. What would you say are the difficulties in recognizing sepsis in your everyday work? Why is the early detection of sepsis difficult in some cases?

<u>Backup questions:</u>

o If you were asked by your supervisor (/ a good colleague) what could be actively done to improve the early detection of septic patients, what would your answer be?

o In your experience, what distinguishes the detection of sepsis from the detection of other (critical) illnesses?

o What increases your confidence in recognizing sepsis?

Acute treatment

We are interested in your experience with the acute treatment of sepsis/septic shock in the course of your daily work. Note: The rehabilitation/aftercare of sepsis survivors will be discussed separately. For this moment, please focus exclusively on acute treatment.

6. How do you perceive or how would you describe the acute treatment process of sepsis?

7. What makes it easier for you personally to treat acutely septic patients in your everyday work? Please explain.

8. What would you say are the difficulties of acute treatment in your everyday work? Why is immediate and rapid treatment difficult in some cases?

Backup questions:

o If you were asked by your supervisor (/ a good colleague) what could be actively done to ensure the immediate and speedy treatment of septic patients, what would your answer be?

o In your experience, what distinguishes the treatment of sepsis from the treatment of other (critical) illnesses?

o What increases your self-confidence in successfully treating sepsis?

Rehabilitation/Aftercare

We are interested in your experience with the rehabilitation/aftercare of survivors of sepsis/septic shock in your everyday professional life.

9. How do you perceive the rehabilitation/aftercare process for sepsis survivors or how would you describe it?

10. What is particularly important to you for the successful rehabilitation/aftercare of a patient after sepsis? What do you think should be taken into account?

11. What makes the rehabilitation/aftercare process easier for you personally in your everyday work?

12. What specific challenges do you face in the rehabilitation/aftercare of sepsis survivors?

Sector transitions

A large number of specialist disciplines and sectors are usually involved in the care of a patient with sepsis/septic shock and a sepsis survivor. We are therefore interested in how you perceive such sector transitions and the associated patient handovers.

13. As a ... you are part of preclinical/internal/postclinical sepsis care. Who usually hands over septic patients (for in-hospital: if the sepsis was not acquired on your ward or nosocomially) or sepsis survivors to you?

14. Describe your ideal admission of a septic patient or sepsis survivor. What requirements must be met for this?

15. What specific steps and information would you like to receive from colleagues in the upstream sector (in the case of preclinicians: relatives and nursing home) when handing over a septic patient or sepsis survivor in order to ensure successful further treatment?
