# Supplementary material for: Healthcare Professionals’ Perspectives on Sepsis Care Pathways—Qualitative Pilot Expert Interviews
Source: J Clin Med. 2025 Jan 18;14(2):619. doi: 10.3390/jcm14020619 (PMC11766067; doi:10.3390/jcm14020619)
Supplement: Supplementary file 1 [file jcm-14-00619-s001.zip › Supplementary SC_Example_quotations.pdf]

Supplementary SC: Example quotations

I → Interviewer

P → Participant

***Supplementary Table S2.* Recognition**

Subcodes	Example quotations
Clinical intuition / clinical view	*That's how I feel, especially with septic patients, it's difficult, you always have the feeling that they're somehow not doing well. They themselves might say: "Oh no, it's better", but you somehow have the medical instinct or whatever you want to call it, that the patient is somehow not well.* (participant No. 2)
Vital parameters	*We also have the patients on the monitor. And of course, if a patient suddenly goes into atrial fibrillation or has a change in consciousness, this can always be a differential diagnosis of sepsis. But I would say that the trigger, so to speak, why a patient comes into focus is a clinical manifestation that is in some way associated with a change in vital function or vital signs.* (participant No. 5)
Lab results	*Then, of course, the laboratory diagnostics will come at some point, then it is relatively simple. If the inflammation values are elevated and perhaps the PCT is also elevated, then it is more likely to be sepsis.* (participant No. 2)
Disease awareness	*I: So what practical steps need to be taken to ensure that treatment can be carried out quickly and efficiently? P: Well, the most important thing, of course, is that this / [...] The awareness! That awareness is raised. That was / Well, I grew up at a time when the clinical picture of sepsis was neither taught nor made clear to anyone in any other way. I only learned about it / When was that? Maybe 2005. 2005/6 was the first time I really heard that the disease existed and how important it was to treat it quickly. Before that, it didn't really exist in my world. Probably all my other colleagues didn't either, otherwise I would have heard about it. So awareness is probably one of the most important things [...].* (participant No. 3)
Clear symptoms	*Or you just have clear symptoms that really tip you off, so if there is a sudden deterioration in general condition when urinating, then the urosepsis is not far away. Or pneumonia, if they [the patients] are tachypneic and have sputum, then it [the recognition of sepsis] is not so difficult.* (participant No. 2)
Correct triage	*And that, I think, is a very, very big task for the emergency department, to make the right disposition. Of course, you could say that the patient was fine at the time of transfer and was fit to be admitted to a normal ward. But often the wrong decisions are made. This is reflected in the fact that two hours later the patient was admitted to the intensive care unit because he was much worse. [...] So you shouldn't overtriage and admit everyone to intensive care because the resources aren't there, but selecting the right patients who will benefit from intensive care monitoring or therapy is the difficult part.* (participant No. 2)
Experienced nursing staff	*Until recently, it has to be said, we had a very high proportion of nursing staff with many years of experience, who are to a certain extent the key to the early detection of septic processes.* (participant No. 5)

SOPs, algorithms, acronyms, scores	*So I think the scores speed things up. That you at least recognize: "This is not just a simple infection that can perhaps still be treated on an outpatient basis, but we are now talking about a septic clinical picture."* (participant No. 2)<br><br>*I: Could you tell me what makes it easier for you personally to recognize sepsis patients as septic at an early stage? [...] P: For me, it's actually a really comprehensive whole-body ABCDE scheme. That you take all the findings from head to toe. [...] And then really a complete ABCDE scheme, on the basis of which I can then make a really good assessment: tachypnoeic patient, tachycardic patient, hypotensive and even a / as difficult as it is to undress the patient as far as possible in order to see a complete inspection of the body and then recognize signs of reduced perfusion.* (participant No. 1)
Trainings	*That's why it's crucial that the nursing staff in particular and the young medical colleagues on the normal wards, who also do the night shifts outside of the core working hours, where there are specialists and senior physicians, don't let this slip through their fingers, that they are trained in clinical recognition features, so that the staff know what to look out for.* (participant No. 5)

Supplementary Table S3. **Treatment**

**Subcodes**	**Example quotations**
High workload	*I: [...] So what is the reason in a large number of cases that acute treatment cannot be initiated as quickly as desired? [...] P: Then it's clearly the work overload. There's no question about it: when there's too much to do and you simply can't take care of the work.* (participant No. 3)
Unknown origin of infection	*But the barrier to say: "We'll give antibiotics immediately", even if you perhaps don't even know the focus yet or if you don't even know why the patient is in shock - in my opinion, there is still a very high barrier.* (participant No. 2)
Wealth of experience and qualification	*And that many, so to speak, hopeful therapeutic strategies have failed somewhere in the end, that the magic bullet does not exist, that a high degree of individualized, pathophysiologically oriented measures are required with, let's say, a certain background of experience in order to adequately care for such complexly ill patients in an acute situation and, above all, not to harm them.* (participant No. 5)
Fixed diagnosis	*[...] but overall, I have to say, my feeling is that once the diagnosis has been made, the therapy is actually initiated well and quickly.* (participant No. 3)
Multiple-eye-principle / low-threshold communication	*And that's why the principle of multiple eyes always applies, so to speak. So if anyone notices anything, that perhaps something has been forgotten, then always raise your finger and say: "You, I've noticed that here, I don't know / What about hydrocortisone or something? [incomprehensible] Or when do we want to start with vasopressin?" So I think that's what I mean, that there has to be a kind of team competence that is not based on hierarchical structures, but ultimately on the fact that everyone basically has a part to play in the care of the patient and that every voice counts in some way. So even when it comes to asking critical.* (participant No. 5)<br><br>*Good communication with each other is relatively important here. Because there are always comments from the side, some of which make you think again: could we do this, could we do that? These are the kinds of things that doctors are happy to take on in order to simply optimize the process.* (participant No. 4)

***Supplementary Table S4.* Rehabilitation/Aftercare**

**Subcodes**	**Example quotations**
Availability of primary care actors	*And some patients can do it all themselves [organizing support] and can take care of it, but as I said, if they don't get a physiotherapist or don't get a care service, that's actually almost worse. These are always the things that are difficult in the home and even more difficult in the countryside because the journeys are even longer and there is often no physiotherapy practice or anything like that in a village. That's quite a journey.* (participant No. 8)
Organizational/ coordinative challenges	*And then it's often a bit of a logistical problem for me as a GP. Do I need a nursing service? How are they looked after? What medication do they need? Do they get anything from the hospital? How quickly have I written a prescription and supplied them? Do I perhaps have my, I mean a non-medical practice, so can I send them there already? What is actually important then? How do I organize that? And that's sometimes more difficult than the medical side of things. That's my experience.* (participant No. 7)
Initial primary care organized by other providers (after clinic discharge)	*It is a relief if something has already been organized during the hospital stay, by the social services, by the hospital. For example, if they have already applied for rehab or at least organized it. If aids have already been prescribed. That when the patient comes home, it's already there: the care bed or the aid or the rollator or the walking stick [incomprehensible]. And that the hospital has already provided initial care, for example through home nursing care. So that the first few days are bridged. That a few medications have already been given. That makes it [the aftercare process] easier for me if it's been organized in advance.* (participant No. 7)<br><br>*So when the patients come home of course and already have a certain set of medication and dressing materials at home, that alone is, we're not under so much pressure that we then / When they are discharged on Friday noon, that we then have to get that [medication] on Friday evening. But that's really / So that's really rare. Almost all clinics already have that / They have to be allowed to write the prescription. Exactly, that's very important. That it's practical / That they [patients] already have something [medication] at home. But it's quite often the case that they come to me on Mondays and they have been home since Thursday and then they haven't taken anything [medication] for a few days because they haven't had it. That also happens.* (participant No. 8)
Transfer management	*I: [...] are there any conditions or circumstances that make the further treatment process after the acute phase easier and particularly smooth for you personally? P: Yes, it would be quite banal, the so-called transfer management. But / that patients with a condition after sepsis are then adequately registered in the subsequent facility, which sometimes works well, but not always.* (participant No. 6)

***Supplementary Table S5.* Patient transitions**

Subcodes	Example quotations
Advance notice of a patient’s arrival	*So it is always ideal if I am informed in advance [about the patient’s arrival], shortly before the patient is discharged. First of all / especially so that I know: “Aha, what exactly did the patient have?“ and brief information about the inpatient course and what the patient’s current condition is and what current therapies are now, and that would ideally be shortly before discharge. One or two working days before the patient is discharged, so that I can read up on this and have a look: When do I call him in? When can he come in? And I can get an idea in advance and prepare a bit. That is / would be the ideal transition from the hospital to us to the GP.* (participant No. 7)
Clinical history/preliminary findings/epicrisis/future roadmap	*Yes, well, of course you need the epicrisis, the report, the course of the illness from the clinic. And what other deficits are there? What is important? What medication does he still need to take? Which therapies should be continued and which check-ups? What else is necessary for the patient? This is actually the most important information we [the general practitioners] need or would like to have.* (participant No. 7)
Standardized handover	*As a rule, there is a pre-registration by fax, if necessary to our occupancy department, if necessary a doctor-to-doctor consultation for special occasions. And then the patient’s documents are sent along with the patient. Ideally, there is another contact on the day of the transfer, where the current condition is discussed again. Everyone is informed. The means of transport can adjust. We can adjust to it. [...] / such a standardized procedure via the ambulance service would be ideal.* (participant No. 6)<br><br>Oral and written: *I would like it [handover information] to be written down as well as verbal. Because, of course, handovers are always a sticking point where information is lost, both in the intensive care unit and in the handover from the peripheral to the intensive care unit. It would be good to have written information or to be able to read it again. But also the verbal information, where you can ask questions again.* (participant No. 4)<br><br>*Then a brief description is given of who the patient is. So formally introduced. Then why the emergency services were called, what happened, when it happened, give a timeline, any measures that have already been initiated by the nursing staff, whether there was any medical contact and then preferably en bloque, preferably in writing.* (participant No. 1)
